# Sensitivity Improvement of Phi-OTDR by Fiber Cable Coils

**DOI:** 10.3390/s21217077

**Published:** 2021-10-26

**Authors:** Konstantin V. Stepanov, Andrey A. Zhirnov, Kirill I. Koshelev, Anton O. Chernutsky, Roman I. Khan, Alexey B. Pnev

**Affiliations:** 1 Bauman Moscow State Technical University, 2-nd Baumanskaya 5-1, 105005 Moscow, Russia; a.zh@bmstu.ru (A.A.Z.); koshelev-k@yandex.ru (K.I.K.); chernutsky.a@bmstu.ru (A.O.C.); khan.roman.igorevich@gmail.com (R.I.K.); pniov@bmstu.ru (A.B.P.); 2 Kotelnikov Institute of Radioengineering and Electronics of RAS, Mokhovaya 11-7, 125009 Moscow, Russia

**Keywords:** fiber optic sensor, distributed fiber optic sensor, phi-OTDR, sensitivity improvement

## Abstract

We present a theoretical and experimental study in which we increased the sensitivity of a phase-sensitive optical time-domain reflectometer (phi-OTDR). This was achieved by constructing coils in the sensor cable, which increased the total amplitude of the impact on the fiber. We demonstrate this theoretically using the example of a phase-sensitive reflectometer model and practically in testing grounds with a buried nearby conventional sensor and a sensor with coils. The sensitivity increased 2.2 times. We detected 95% of events when using coils, versus 20% when using a straight cable. The suggested method does not require any modifications to the device.

## 1. Introduction

Distributed fiber-optic systems (FOSs) are used in a large number of areas for recording acoustic impacts: in seismometry, including for oil wells [1,2,3,4,5,6,7]; for monitoring activity near long objects, such as perimeters and pipelines [8,9,10]; and for monitoring railway tracks and transport [11,12] as well as structures, including engines, buildings, and composite airplanes.

Scientists at the University of Texas under the leadership of GF Taylor introduced FOSs based on phase-sensitive reflectometry in the early 1990s [13,14,15]. Over the past years, many studies have been carried out on these systems in various directions, including signal recognition [9,16,17], increasing the range of the controlled area [8,18,19,20], and increasing the system’s spatial resolution [21,22,23].

One important parameter of such FOSs is their acoustic sensitivity threshold, that is, the minimum acoustic or vibration impact that the system can register. Several methods have been proposed to increase the acoustic sensitivity of FOSs, such as the use of a special cable with improved acoustic sensitivity [24,25,26,27,28], several photoreceivers together with special data-processing algorithms [29,30], and a special optical fiber with weak fiber Bragg gratings (wFBGs) recorded in a fiber with a known periodicity [31,32,33,34]. One possibility for increasing the SNR was investigated by using two probe pulses simultaneously [35,36,37,38], including with a certain polarization configuration [39,40].

In this work, we study another way to increase the acoustic sensitivity of a phase-sensitive optical time-domain reflectometer (phi-OTDR). Its advantages include simplicity and lack of need to upgrade any devices. The effect is achieved by using fiber coils in the cable in certain places or with some periodicity. This method of increasing the acoustic sensitivity does not exclude other existing methods and can be used together with them.

## 2. Theory

**Figure 1:**
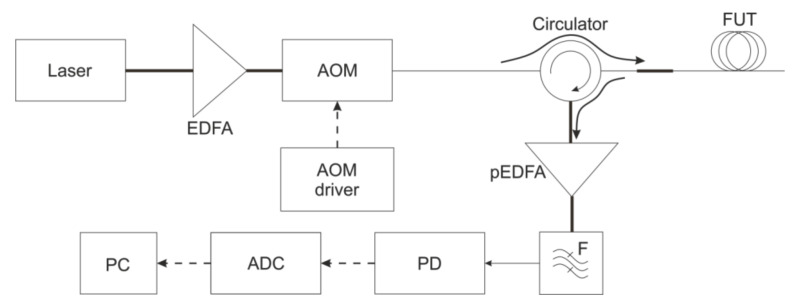
Phi-OTDR schematic.

A phi-OTDR is a device that can detect acoustic impacts on a cable based on Rayleigh backscatter analysis. The schematic of the device is shown in Figure 1. The radiation source is a frequency-stabilized narrow-linewidth laser, with a coherence length that is much greater than the pulse half-width (*τ_pulse_*~10...500 ns). This causes backscattered radiation interference in each pulse position. The continuous radiation from the laser is amplified by an erbium-doped fiber amplifier (EDFA). After the acousto-optic modulator (AOM) modulates the radiation to probing pulses, it goes to the sensor fiber through the circulator in the forward path. The backscattered radiation passes in the opposite direction in the circulator. Then, it is amplified by the pre-EDFA. Its amplified spontaneous emission (ASE) is excluded after it passes the narrow optical filter. Then, radiation enters the photodiode (PD) and is digitized on the analog-to-digital converter (ADC) before processing on the personal computer (PC).

**Figure 2:**
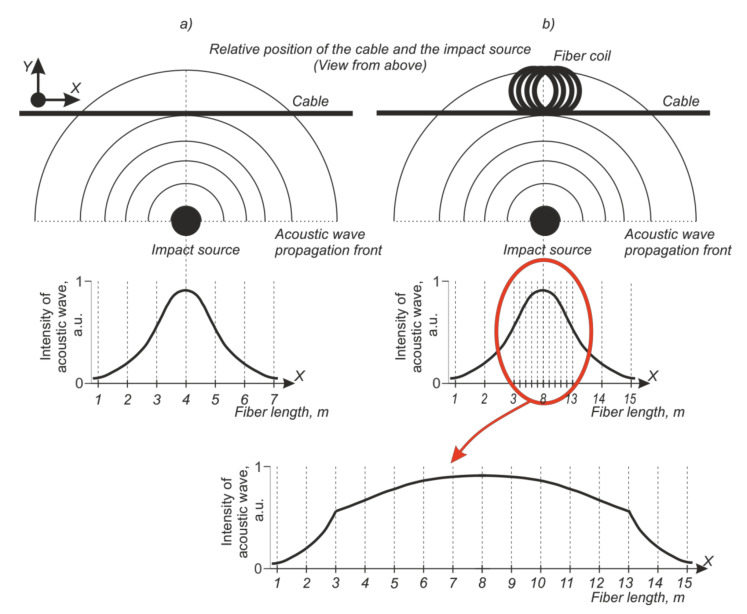
Diagram of the acoustic signal propagation for straight (**a**) and coil (**b**) configurations.

Due to the long coherence length of the radiation source on the phi-OTDR’s PD, the amplitudes of the backscattered waves are summed, taking into account their phases. For one probe pulse, the addition occurs over a section whose length is equivalent to the half-width of the pulse duration $\left(\Delta z=\frac{1}{2}\cdot \frac{c}{n}\cdot \tau_{pulse}\right)$, which is equal to the system’s spatial resolution, where *c* is the speed of light, and *n* is the effective refractive index of fiber core. An acoustic wave from an external source decays with distance. If the wave reaches its maximum amplitude at each point of the fiber with a length of Δz, then attenuation due to the section length will not occur. The acoustic sensitivity will increase due to the fiber coils, since the amplitude of the impact over the entire interval of the spatial resolution does not decrease. This increases the impact’s contribution on the resulting interference signal. The method’s operating principles are demonstrated in Figure 2 for simulated data. Figure 2a shows how the intensity of fiber deformation depends on the cable’s coordinates and the distance from the impact site. Figure 2b shows a similar change in intensity when the cable is laid with a coil. Obviously, for the same cable length as in the first case, the greater level of impact is total. For example, compare the square under 1–7 m in graph 2a with that under 5–11 m in Figure 2b. Thus, the signal produced by the device also should increase in intensity.

Notably, in this case, it is necessary to match the fiber’s length with the coordinates of the controlled object since the cable’s physical length will also contain the length of the fiber spools.

**Figure 3:**
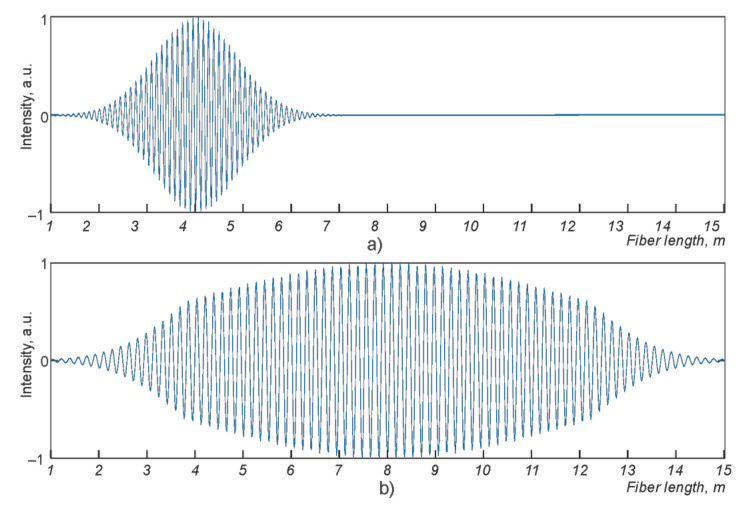
Plots of scattering center shift distributions along the cable for straight (**a**) and coil (**b**) configurations.

If the reflecting centers on the fiber section for the two cases under consideration are displaced, then the attenuation of the amplitude along the length of the sensor will change. An example of the resulting picture of a simulation after filtering is shown in Figure 3.

**Figure 4:**
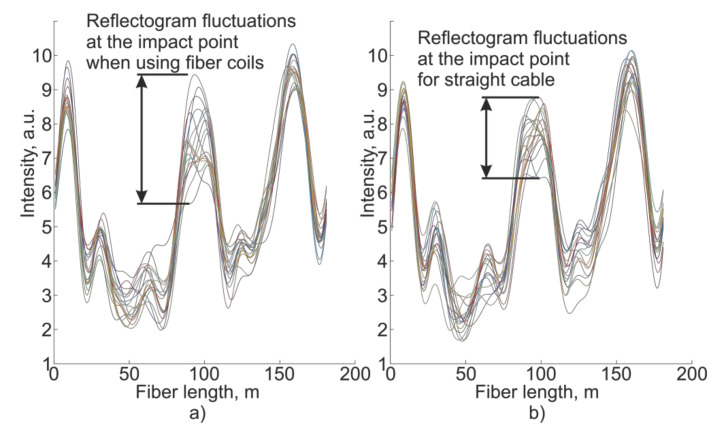
Reflectogram fluctuations for the sensor with (**a**) and without (**b**) fiber coil.

**Figure 5:**
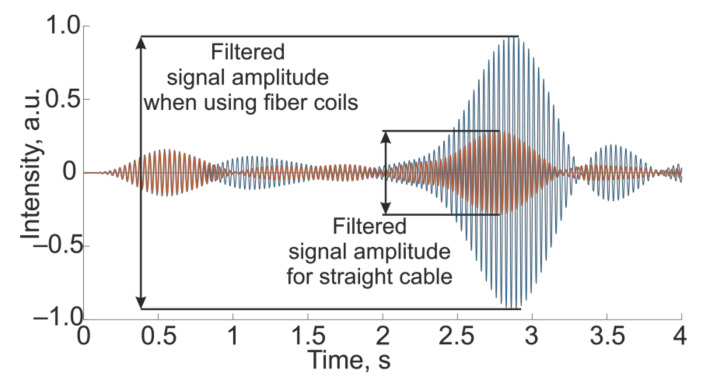
Filtered modeled signal for straight cable and fiber coils.

This change in the signal response will increase the impact’s contribution in the data recorded by the receiver (Figure 4), which will increase the amplitude. Following the operating principles of a phi-OTDR, it is convenient to check this using the filtered signal, as shown in Figure 5.

Fiber coils can be used for other applications than signal enhancement at a certain point. Some applications do not require using the full available length of the phi-OTDR sensor, e.g., perimeter monitoring of an object or a short pipeline that has a length less than 20 km. The user only needs 20 km or less out of the usual 50 km length of a phi-OTDR. In such conditions, it is possible to create coils from cable length equal to the spatial resolution ∆*z* of the phi-OTDR with the same ∆*z* period between them in the buried trench. In such configurations, a fiber with length *L* will be used for a sensor with length *L*/2. Coils can be created on the entire sensor length or only in some special and important zones to enhance sensitivity.

## 3. Modeling

For theoretical analysis of the proposed method, a mathematical model was developed of the signal-formation process in a fiber-optic sensor system based on a phase-sensitive reflectometer with fiber coils. It is interpreted using the following considerations [41]. The amplitude for a point with the *x* coordinate along the cable undergoes attenuation with a coefficient α, and it also has the probability density of a Gaussian distribution, as determined by Formula (1):

(1) $$A\left(x\right)=A_{0}\cdot exp\left(-2x\alpha \right)\cdot p\left(a\right),$$

where $p\left(a\right)=\frac{1}{\sigma \sqrt{2\pi }}exp\left\{-\frac{{\left(a_{0}-a\right)}^{2}}{2\sigma^{2}}\right\}$ is the probability density of a Gaussian distribution, and *σ* is the scale parameter.

The radiation source is not ideal and has instability over time. The array of values ν for the radiation frequency source within the interval of simulated time interval T at each moment is described by Equation (2):

(2) $$\nu =\nu_{0}+\left|F^{-1}\left\{\sqrt{S_{\nu }\nu_{p}{}^{2}T}\right\}\right|,$$

where $\nu_{0}$ is the laser frequency at the initial moment, in Hz;$\;F^{-1}\left\{\;\right\}$ is the inverse Fourier transform; $S_{\nu }={\hat{S}}_{\nu }\cdot {\left|F\left\{rand\left(1\right)\right\}\right|}^{2}\frac{1}{\nu_{p}{}^{2}T}$ is the laser frequency’s instability, as determined by the envelope ${\hat{S}}_{\nu }$;$\;\nu_{p}$ is the the pulse repetition rate (in our case, 1 kHz); T is the duration of simulated time, in s; and $F\left\{\;\right\}$ is the Fourier transform.

The radiation detected by the receiver has a phase that depends on the source wavelength λ = c/ν and the distance to point *x* along the cable and is determined by Formula (3):

(3) $$\Theta \left(x\right)=\frac{4\pi n\left(x+\Delta x\right)}{\lambda },$$

where *n* is the effective refractive index of the fiber core, and Δ*x* is the coordinate displacement due to external influence, in µm.

In the simulation, a shift of the scattering centers was specified, similar to the shift of a fiber section wound around a piezoelectric cylinder. The simulation was carried out for various values of the maximum shear at the end of the piezoelectric $\Delta L_{max}$ with the frequency *ν_ac_*. The impact is described by Equation (4):

(4) $$\Delta x\left(l,t\right)=\left\{\begin{array}{c} \Delta L_{max}\cdot \frac{l}{l_{max}}\cdot sin\left(2\pi \nu_{ac}t\right)\\ 0\;,otherwise \end{array}\right.,\;\left\{\begin{array}{c} 0\leq l\leq l_{max}\\ t\in t_{impact} \end{array}\right..$$

Thus, the complex amplitude from each point, taking the phase into account, is determined by Equation (5).

(5) $$E\left(x\right)=A\left(x\right)\cdot exp\left(i\Theta \left(x\right)\right),$$

where *i* is the imaginary unit.

For sensors based on phi-OTDR, the receiver detects the interference result of all backscattered waves at half-pulse width *τ_pulse_*/2, which can be written according to Equation (6):

(6) $$E_{sampling}\left(x\right)=\overset{x+\frac{\tau_{pulse}}{2}\cdot \frac{c}{n}}{\underset{x}{\int }}E\left(\bar{x}\right)d\bar{x}.$$

In addition to interference, the receiver has a transfer function, as defined by Equation (7).

(7) $$Mask\left(\upsilon \right)=exp\left(-{\left[\frac{\upsilon }{\upsilon_{PD}}\right]}^{2}\right),$$

where *υ_PD_* is the receiver’s bandwidth.

By adding the receiver’s transmittance function, expressed according to Formula (7), to the resulting intensity described by Formula (6), we obtain Expression (8), which determines the intensity recorded by the receiver for each point at coordinate *x* along the cable. We obtain Formula (9) by writing out all of the components in this expression:

(8) $$I_{Receiver}\left(x\right)={\left|E_{sampling}\left(x\right)\right|}^{2}⊗F^{-1}\left\{Mask\left(\upsilon \right)\right\},$$

where ⊗ is the convolution operator.

(9) $$I_{Receiver}\left(x\right)={\left|\overset{x+\frac{\tau_{pulse}}{2}\cdot \frac{c}{n}}{\underset{x}{\int }}A_{0}\cdot exp\left(-2\bar{x}\alpha \right)\cdot p\left(a\right)\cdot exp\left(i\frac{4\pi n\left(\bar{x}+\Delta x\right)}{\lambda }\right)d\bar{x}\right|}^{2}⊗F^{-1}\left\{exp\left(-{\left[\frac{\upsilon }{\upsilon_{PD}}\right]}^{2}\right)\right\}.$$

The output data after the simulation were the SNR values for various input parameters, including the impact amplitudes. The SNR was calculated as follows.

After simulation, the original signal passed a narrow-band filter corresponding to the frequency of the impact. The algorithm used a 10th-order Butterworth bandpass filter with a 3 dB width of less than 5 Hz from the central frequency of exposure.

The standard deviation over time interval Δ*t* (for each sensor point) was considered without influence (*t*∉*t_Imp_*). This value was equal to the *RMS_noImp_* parameter.

For the entire simulation time, the standard deviations (SDs) were found for time sections Δ*t*:

SNRSDi=SDiSDnoImpSNR=maxSNRSDi

**Figure 6:**
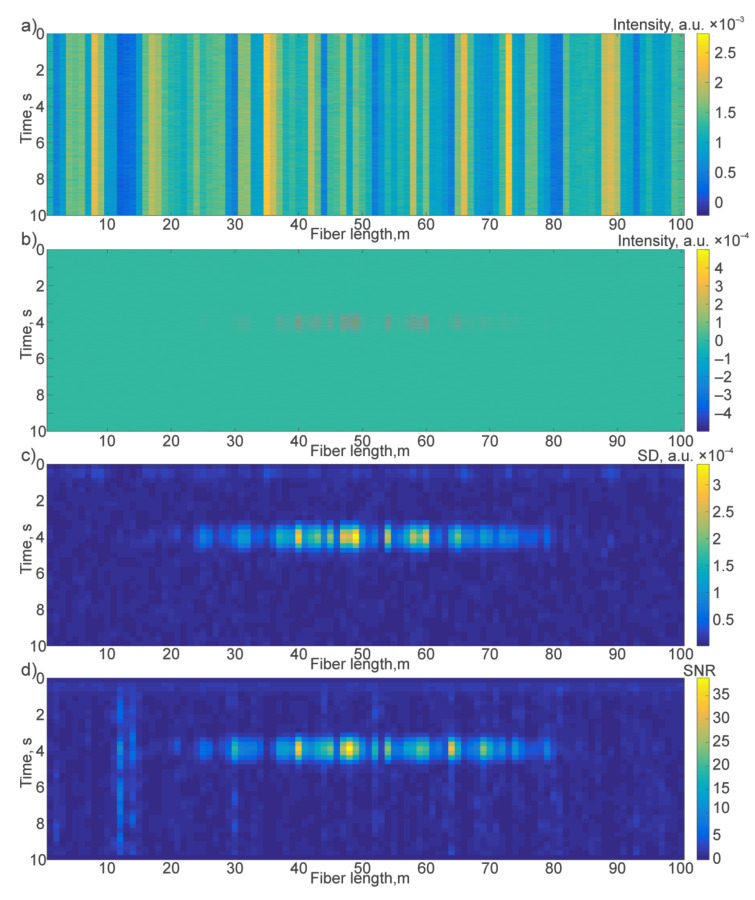
View of the data at different stages of the algorithm: (**a**) raw data with 1 kHz discretization; (**b**) data after bandpass filtering; (**c**) filtered data of standard deviations in 500 ms windows with 250 ms steps; and (**d**) the final SNR with 250 ms steps.

The data-processing stages from raw data to the final SNR values are shown in Figure 6.

Due to the system behavior’s random nature, the value of *SD_noImp_* can vary and have different values, even for areas where there is initially no impact. Because of this, the *SNR_SD_* can differ, and *SNR_SD_* > 1 in any region does not mean that there has been an impact. In further modeling, the condition chosen for stable recognition of an impact was when the largest value in the array *SNR_SD_(i)* ≥ 5.

**Figure 7:**
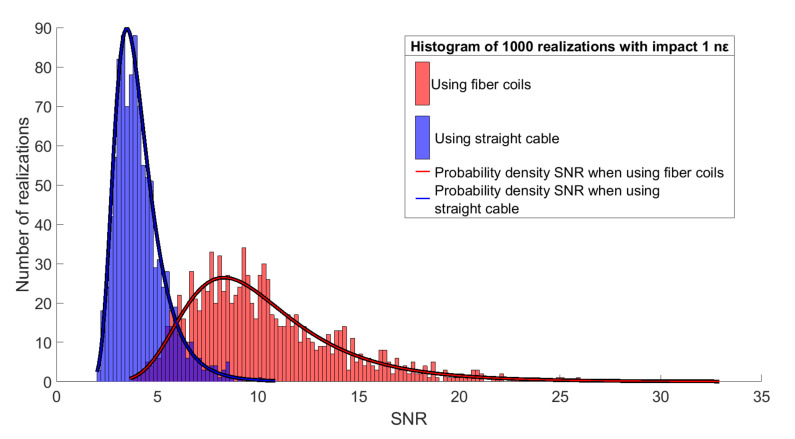
Histogram plots of SNR values for straight cable and fiber coil.

For a more accurate assessment of the system, 1000 realizations were simulated for each amplitude of the impact. A simulation was carried out for two cases: a classic cable (attenuation of the impact immediately from the epicenter) and an increased length of optical fiber corresponding to the system’s spatial resolution. In this case, an impact was simulated, and the classic cable showed amplitudes significantly lower than the threshold of 1 nε. Distribution histograms of the obtained SNR values are shown in Figure 7.

Table 1 compares the accuracy parameters between the straight cable and fiber coil methods.

The fiber coil method allowed the simulated impact amplitude parameters to increase the SNR by 2.5 times, which increased the detection probability to 95%.

## 4. Field Tests

**Figure 8:**
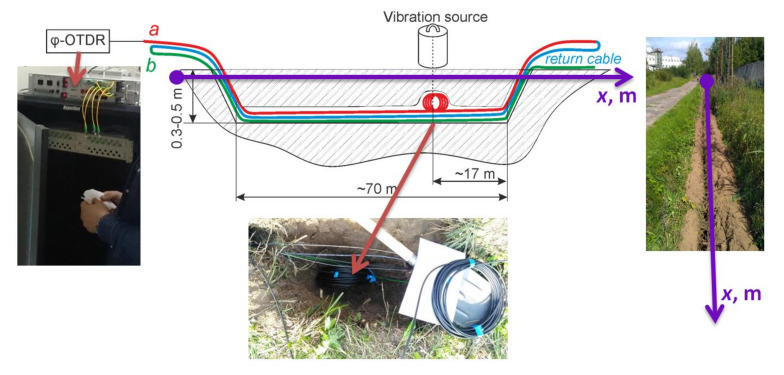
Diagram and photographs of the test setup.

An experimental study of the proposed method based on fiber coils was carried out on the BMSTU test site. The optical fiber cable was buried in a 30 cm deep trench, which corresponds to the standard cable laying depth for DAS. In the experiment, we used one brand of optical cable in different laying configurations. The experimental setup is shown in Figure 8.

The fiber cable configuration comprised a) a cable with a 10 m long fiber coil, corresponding to the system’s spatial resolution of the system during the experiment, and b) a straight cable.

Table 2 shows the phi-OTDR parameters used for the experiments.

The reasons for such parameter values were as follows. Frequencies of impacts in the vicinity of 38 Hz are common for recording events such as human passes and digging, since this range does not contain low-frequency noise from environment sources and laser wavelength deviation but is still just slightly attenuated in the soil. Frequencies in the region of 55 Hz were used for searching steps via fiber cable buried in rocky soil at a shallow depth. A frequency of 101 Hz was investigated as the cutoff frequency of sound propagation in the soil, above which strong attenuation begins. The distances to the vibration source were chosen according to requirements in monitoring systems for detecting steps using distributed acoustic sensors. The source of the vibration impacts had an uneven frequency response: at a frequency of 38 Hz, the impact’s amplitude was about 5 times higher than at an impact frequency of 101 Hz.

**Figure 9:**
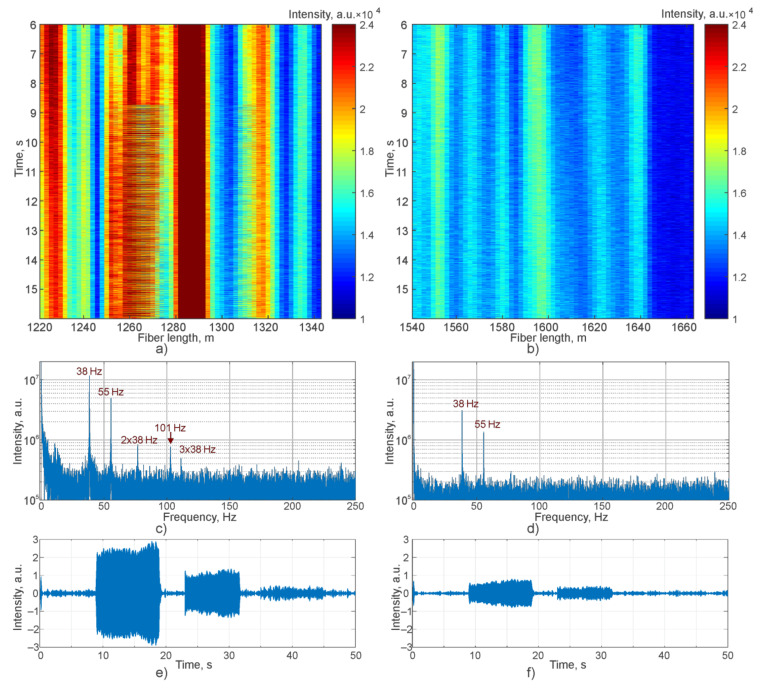
Experimental data: (**a**) raw data waterfall of vibration start for the coil point (1270 m); (**b**) raw data waterfall of vibration start for the straight cable point (1570 m); (**c**) signal spectrum for the 1270 m point; (**d**) signal spectrum for the 1570 m point; bandpass-filtered data during the entire experiment at the 1270 m (**e**) and 1570 m (**f**) points.

Data obtained during the experiment are shown in Figure 9. Raw waterfalls in time duration from 6 to 16 s of the experiment represent the vibration start at the coil point (1270 m) and at the straight cable point (1570 m) in Figure 9a,b, respectively. Figure 9c,d show the signal spectra at marked points. For the point with coil, all impact frequencies and a few harmonics for 38 Hz are visible. For the straight cable only 38 Hz and 55 Hz peaks are observable. Figure 9e,f show bandpass-filtered data for the marked points during the entire experiment.

The obtained experimental data confirmed the rapid degradation of weak vibration impacts as the point of influence moves away from the cable [42]. The vibration influence at a 38 Hz frequency, which had a higher power (due to the source’s uneven characteristics), together with a lower attenuation coefficient in the ground, also underwent attenuation with distance but was successfully recorded in all of the experimental configurations.

**Figure 10:**
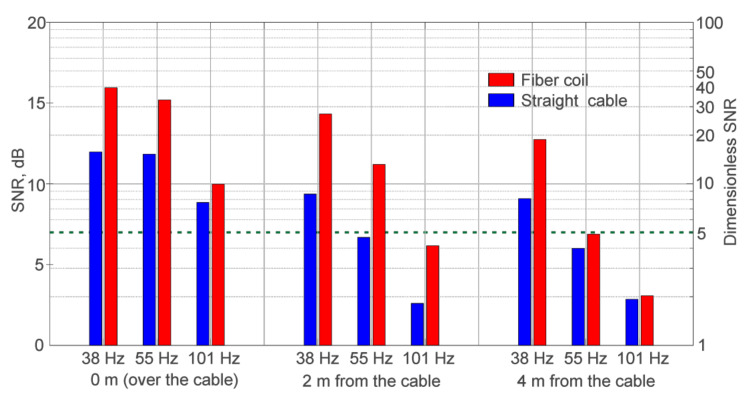
Plot of experimental SNR values.

Table 3 and Figure 10 show the SNR values obtained for each experiment for cables a and b.

As can be seen from the obtained results, the use of the fiber coil made it possible to increase the SNR by an average of 2.2 times. The obtained results were consistent with the indicators calculated in the modeling, with a difference of 12%. The signal formation process in a phi-OTDR depends on many factors and its intensity has a Rayleigh distribution. As a consequence, it influences the signal intensity level and sensitivity of the device. In computer modeling, we are able to simulate a large number of realizations and obtain a histogram of possible SNR values, which is similar to the Rayleigh distribution. But it is difficult to collect the same number of data realizations in a field-test experiment since it takes significantly more time and resources. This is the reason for the deviation between SNR improvement in the simulation and in the experiments. However, even with the number of experiments performed, the values obtained are quite similar to the modeled data and allow the model estimate to be considered correct.

## 5. Discussion

The paper considered how fiber coils in cable affected the acoustic sensitivity of a phi-OTDR. To confirm the method’s effectiveness, mathematical modeling was conducted for two types of systems: one with a straight cable and one with fiber coils. With a low impact amplitude, which a classic cable registered in less than 20% of cases, fiber coils increased the SNR by 2.5 times, which increased the detection probability to more than 95% for 1000 realizations of the simulation system. An experimental study with fiber coils, with length corresponding to the system’s spatial resolution, also confirmed the method’s effectiveness, and the obtained results showed that SNR increased by 2.2 times.

## Figures and Tables

**Table 1:** Accuracy parameters of different cables.

Parameter	Straight Cable	Fiber Coil Method
Distribution mode, SNR	3.45	8.37
Number of realizations with SNR < 5	807	21
Detection probability	19.3%	97.9%

**Table 2:** The phi-OTDR parameters used for the experiments.

Parameter	Value
ADC	50 MHz (the equivalent of 2 m spatial discretization)
Pulse duration	100 ns
Spatial resolution	10 m
Impact frequencies	38 Hz, 55 Hz, and 101 Hz
Distance from the vibration source to the cable	0 m (over the cable), 2 m, and 4 m

**Table 3:** Experimental SNR values.

	Straight Cable	Fiber Coil	
Mean dimensionless SNR at 38 Hz (Number of experiments with SNR > 5)			Dimensionless SNR increase
0 m (over the cable)	15.75 (10 of 10)	39.37 (10 of 10)	2.50
2 m from the cable	8.64 (7 of 10)	27.09 (10 of 10)	3.13
4 m from the cable	8.09 (9 of 10)	18.8 (10 of 10)	2.25
Mean dimensionless SNR at 55 Hz (Number of experiments with SNR > 5)			
0 m (over the cable)	15.27 (10 of 10)	33.03 (10 of 10)	2.16
2 m from the cable	4.66 (5 of 10)	13.18 (10 of 10)	2.83
4 m from the cable	3.98 (1 of 10)	4.88 (5 of 10)	1.23
Mean dimensionless SNR at 101 Hz (Number of experiments with SNR > 5)			
0 m (over the cable)	7.66 (7 of 10)	9.96 (9 of 10)	1.30
2 m from the cable	1.82 (0 of 10)	4.14 (3 of 10)	SNR < 5
4 m from the cable	1.93 (0 of 10)	2.03 (0 of 10)	SNR < 5

## Data Availability

The data presented in this study are available on request from the corresponding author.
